# A Brachytherapy Plan Evaluation Tool for Interstitial Applications

**DOI:** 10.1155/2014/376207

**Published:** 2014-02-09

**Authors:** Surega Anbumani, N. Arunai Nambiraj, Sridhar Dayalan, Kalaivany Ganesh, Pichandi Anchineyan, Ramesh S. Bilimagga

**Affiliations:** ^1^Department of Radiation Oncology, HCG Bangalore Institute of Oncology, RRMR Extension (KH Road), Bangalore 560027, India; ^2^Photonics, Nuclear and Medical Physics Division, VIT University, Vellore 632014, India

## Abstract

Radiobiological metrics such as tumor control probability (TCP) and normal tissue complication probability (NTCP) help in assessing the quality of brachytherapy plans. Application of such metrics in clinics as well as research is still inadequate. This study presents the implementation of two indigenously designed plan evaluation modules: Brachy_TCP and Brachy_NTCP. Evaluation tools were constructed to compute TCP and NTCP from dose volume histograms (DVHs) of any interstitial brachytherapy treatment plan. The computation module was employed to estimate probabilities of tumor control and normal tissue complications in ten cervical cancer patients based on biologically effective equivalent uniform dose (BEEUD). The tumor control and normal tissue morbidity were assessed with clinical followup and were scored. The acute toxicity was graded using common terminology criteria for adverse events (CTCAE) version 4.0. Outcome score was found to be correlated with the TCP/NTCP estimates. Thus, the predictive ability of the estimates was quantified with the clinical outcomes. Biologically effective equivalent uniform dose-based formalism was found to be effective in predicting the complexities and disease control.

## 1. Introduction

Normal tissue complication probability and tumour control probability models have been developed in the past decades with some complexities in their execution [1]. Clinical usage of these models presents a difficulty due to the lack of precise knowledge [1]. Most of these radiobiological models were primarily used as research tools and remain to be clinically validated for the routine use in the clinics. Hence, a number of software tools have been developed in the past decade. BIOPLAN was developed in the visual basic platform by Sanchez-Nieto and Nahum [2]. It can be used to calculate dose response based on Poisson, Zaider Minnerbo, LQ, CV, and SDR models. A computational environment for radiotherapy research was developed by Deasy et al. in 2003 [3]. Warkentin et al. in 2004 have developed a TCP, NTCP_calc tool based on critical volume (CV) and sigmoidal dose response (SDR) models [4]. DREES and EUCLID were the MATLAB-based calculation programs which model the clinical outcomes using multivariate analysis structure [5, 6]. EUD Model, a MATLAB code developed in 2007, calculates the TCP and NTCP with a unified formula [7]. An integrated computational platform for analyzing radiotherapy outcomes was developed in 2009 [8]. An online access tool that used the DICOM RT files as input was developed with MS ACCESS. It could calculate the TCP and NTCP estimations. HART (Histogram Analysis in Radiation Therapy) program calculates a metric called complication free tumor control (p+) using the DICOM RT files as the input [9]. Dose convolution factor and P+ can be calculated in MATLAB platform with SABER (Spatial And Biological Evaluation of Radiotherapy) program that uses the spatial DVH concept [10]. The software tools listed so far are used to calculate TCP/NTCP for any external beam radiotherapy (EBRT). Brachytherapy is an integral part of any radiotherapy procedure followed alone or along with EBRT. There is no exclusive tool to evaluate brachytherapy on the basis of TCP and NTCP. Hence, our aim is to develop a unique external evaluation module to compute TCP and NTCP from the DVH data and other quality metrics of interstitial brachytherapy plans.

**Figure 1:**
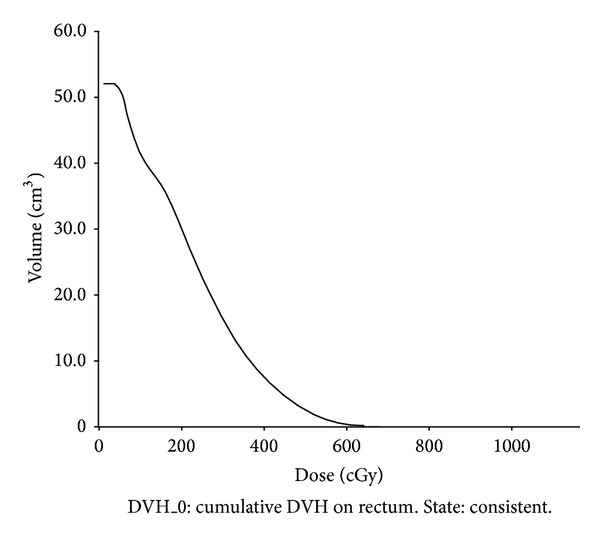
Dose volume histogram rectum.

Treatment planning is a trial and error process that could result in a number of plans. Hence, radiotherapy treatment plans undergo a rigorous evaluation procedure by the doctors and physicists before the actual implementation on patients. Plans are conventionally evaluated using DVH, an inbuilt tool in treatment planning system (TPS). It lacks biological metrics such as tumor clonogen cell density. There may be an inaccuracy inherent in evaluating the clinical outcome without the use of such biological metrics. To achieve the best possible tumor eradication of uniform clonogen cell density, uniform dose distribution within the tumour volume is needed. High dose rate interstitial implants are often characterized by the high dose gradients resulting in nonuniform dose distributions surrounding the applicator insertion. The clinical outcome prediction that is based solely on minimum/mean/median tumor dose would not be appropriate.  Figure 1 shows the rectal dose volume histogram of a patient plan.

## 2. Methods and Materials

### 2.1. Background—The BEEUD Concept

The evaluation tool is constructed to compute TCP and NTCP was based on BEEUD concept, devised by Kehwar et al. [11]. In order to obtain optimal tumor cell killing with uniform clonogenic cell density and to avoid necrosis of the normal cell present within the target volume, the dose distribution within the target volume should be uniform [12]. However, in high-dose-rate (HDR) interstitial implants it is difficult to achieve a uniform dose distribution because of the very high radiation dose in the vicinity of the radiation source. Hence, the tumor control probability (TCP) calculated on the basis of minimum or mean or median target dose would not be appropriate to predict accurate treatment outcome. To solve the problem, an imaginary ideal implant was divided into a large number of voxels to derive the biologically effective equivalent uniform dose (BEEUD) using voxel-based TCP. Then the HDR implant was divided into four different regions, based on the pattern of dose distribution, to define quality indices (QI).

**Figure 2:**
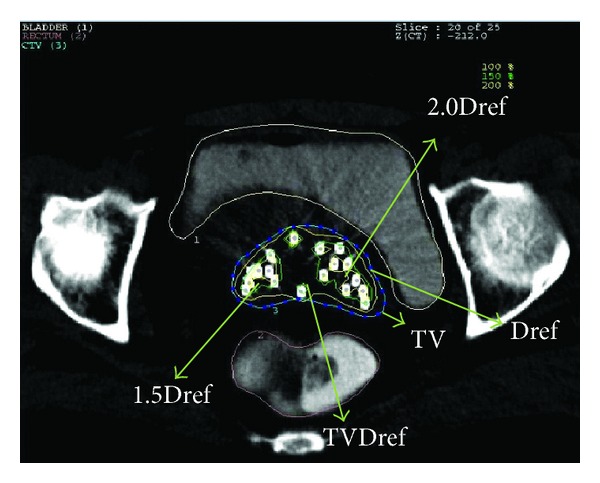
Axial CT slice illustration of dose regions.

The linear quadratic (LQ) model provides a simple way to describe dose response of different fractionation schemes, in terms of the biologically effective dose (BED) [13]. The BED for HDR ISBT [14] for a total dose of *D* (Gy) delivered with dose *d* (Gy) per fraction can be written by

(1) $$\begin{array}{c} \text{BED}=D\left[1+\frac{Gd}{\left(\alpha /\beta \right)}\right], \end{array}$$

where *α*/*β* ratio is the tissue specific parameter and is the ratio of the coefficients of lethal damage to the sublethal damage and *G* is the factor accounting for incomplete repair of sublethal damage during interfraction interval between the fractions. In this study, it is assumed that the time interval between the fractions is sufficient enough to allow the full repair of the sublethal damage; hence, *G* is taken as 1. The tumor control probability (TCP) for uniform dose distribution within the target volume is given by the following formula [15]:

(2) $$\begin{array}{c} \text{TCP}=\exp\left[-\rho V\;\exp\left(-\alpha \;\text{BEDt}\right)\right], \end{array}$$

where *ρ*, *V*, *α*, and BEDt are the clonogenic cell density, target volume, coefficient of lethal damage (radio sensitivity of lethal damage), and BED for the target, respectively. The dose distribution of HDR ISBT within target volume is highly nonuniform and has high dose gradient; hence, the equation cannot be directly applied to compute accurate TCP. To get an appropriate expression of TCP for HDR ISBT implant different regions of HDR ISBT implant have been considered (Figure 2). It is also shown that target volume is divided into four regions:

- region which receives a dose less than reference dose;
- region which receives a dose equal to 1 to 1.5 times the reference dose;
- region which receives a dose equal to 1.5 to 2 times the reference dose;
- region which receives a dose greater than 2 times the reference dose.

The BEEUD and QIs were introduced into the equation of TCP to get an expression for HDR implants.

The final formula for TCP is

(3) TCP=exp⁡⁡[−ρTVDref  ×{[(1−CI)CI]exp⁡⁡(−αBEEUD1)    +DHIexp⁡⁡(−αBEEUD2)    +(DNR−ODI)exp⁡⁡(−αBEEUD3)    +ODIexp⁡⁡(−αBEEUD4)}],

where *ρ* is clonogen cell density, CI is conformity index, DHI is dose homogeneity index, DNR is dose nonuniformity ratio, ODI is overdose volume index, and *α* is radio-sensitivity.

*Coverage Index (CI).* The fraction of the target volume that receives a dose equal to or greater than the reference dose [16] is

(4) $$\begin{array}{c} \text{CI}=\frac{\text{TVDref}}{\text{TV}}. \end{array}$$

*External Volume Index (EI).* The ratio of the volume of normal tissue that receives a dose equal to or greater than the reference dose to the volume of the target [16] is

(5) $$\begin{array}{c} \text{EI}=\frac{\text{NTVDref}}{\text{TV}}. \end{array}$$

*Relative Dose Homogeneity Index (DHI).* This is defined as the ratio of the target volume which receives a dose in the range of 1.0 to 1.5 times of the reference dose to the volume of the target that receives a dose equal to or greater than the reference dose [16]:

(6) $$\begin{array}{c} \text{DHI}=\frac{\left[\text{TVDref}-\text{TV}1.5\text{Dref}\right]}{\text{TVDref}}. \end{array}$$

*Overdose Volume Index (ODI).* This is the ratio of the target volume which receives a dose equal to or more than 2.0 times of the reference dose to the volume of the target that receives a dose equal to or greater than the reference dose [16]:

(7) $$\begin{array}{c} \text{ODI}=\frac{\text{TV}2.0\text{Dref}}{\text{TVDref}}. \end{array}$$

*Dose Nonuniformity Ratio (DNR).* This is the ratio of the target volume which receives a dose equal to or greater than 1.5 times of the reference dose to the volume of the target which receives a dose equal to or greater than the reference dose [17]:

(8) $$\begin{array}{c} \text{DNR}=\frac{\text{TV}1.5\text{Dref}}{\text{TVDref}}. \end{array}$$

Conditions for an ideal implant are where the values of QIs should be as follows:

(9) $$\begin{array}{c} \text{CI}=1,\;\text{EI}=0,\;\text{DHI}=1,\;\text{ODI}=0,\;\text{DNR}=0. \end{array}$$

The target volume and the normal tissue volumes are divided into *n* subvolumes to account for dose heterogeneity in the HDR interstitial implants. Hence, each region with the specified subvolumes has its own biologically effective equivalent uniform dose.

BEEUD_*n*_ Biologically Effective Equivalent Uniform Dose for the 4 regions

NTCP calculation

Normal tissue is divided into two regions:

- region that receives a dose less than the reference dose;
- region that receives a dose greater than or equal to the reference dose.

Consider

(10) NTCP=(NTCPF)k,NTCPF=exp⁡[(N0)−1/k(TVV0)[(VTV−EI)   ×exp⁡{(αk)BEEUDn1}   +(EIV0)exp⁡⁡{(αk)BEEUDn2}]],

where  *N*_0_ is tissue-specific, nonnegative adjustable parameters, *V*_0_ is reference volume of the normal tissue, *k* is tissue-specific, nonnegative adjustable parameter, BEEUD_*n*_ is biologically effective equivalent uniform dose for the 2 regions, where *n* = 1, 2:

- region in which the dose is equal to or less than the reference dose Dref,
- region in which the dose is equal to or greater than the reference dose Dref,

and EI is external volume index.

The two major formulae for TCP and NTCP were split into numerous subvolumes. User friendly input section was also created. The evaluation tool was named as Brachy TCP.m and Brachy NTCP.m to perform TCP and NTCP calculations, respectively, in any personal computer. The MATLAB code could manipulate data in either Excel or DDBS (distributed data base structure) array format. Hence, it is independent of operating systems of TPS.

## 3. Results 

**Figure 3:**
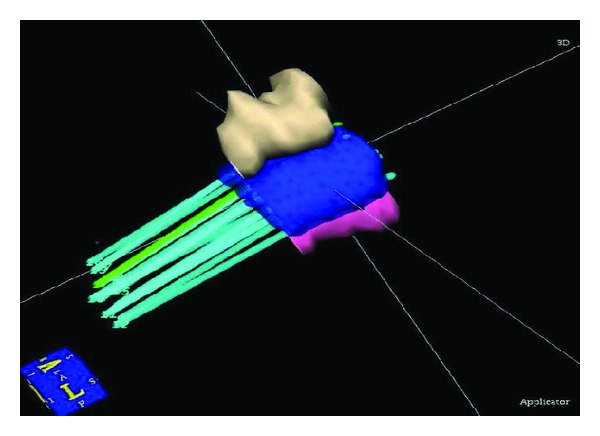
Dose distribution in 3D view.

The clinical target volume, bladder, and rectum were delineated by the radiation oncologists in the patient CT images in the treatment planning system. The needles were then reconstructed and radioactive source loading pattern was simulated. Then the dose was calculated and optimized. A three-dimensional view of the dose distribution is shown in Figure 3.

**Figure 4:**
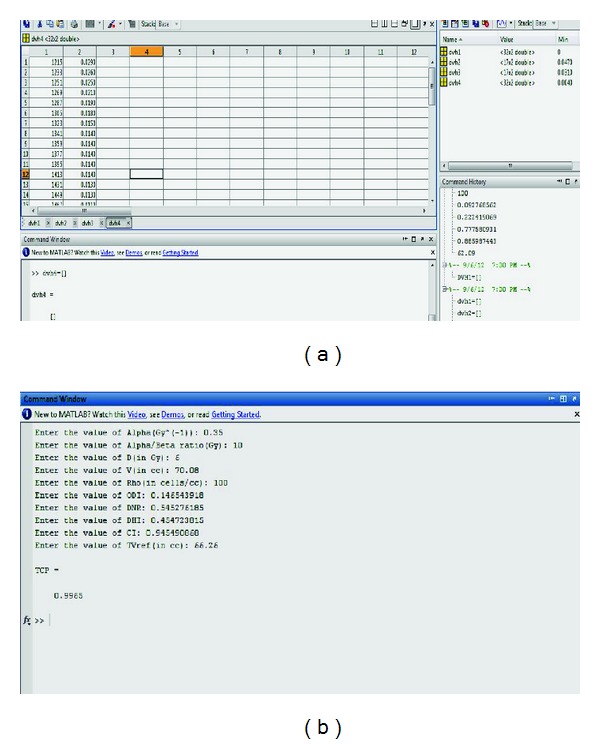
(a) Input section for DVH data. (b) QI input and TCP estimation.

The necessary steps to be followed for executing the program are explained as follows:

- raw data export is exporting the patient DVH file from the TPS to windows-based PC;
- file format, for example, UNIX DVH file in case of Nucletron PLATO TPS, is converted to a user readable EXCEL format (Table 2);
- differential DVH data (*X*-*Y* Coordinates) are segregated into different regions (four for target volume and two for normal tissues);
- in the MATLAB command window two column matrix variable inputs are created by typing
   dvh1 = []
   dvh2 = []
   dvh3 = []
   dvh4 =[]
   For TCP calculation; (Figure 4(a))
   dvh1 = []
   dvh2 = []
   for NTCP calculation;
- the program files BrachyTCP.m/BrachyNTCP.m are executed;
- now a series of questions would appear in the screen for the input of *α*, *α*/*β*, *D*, *V*, *ρ*, ODI, DNR, DHI, CI, and Tvref to calculate TCP for the particular treatment plan (Figure 4(b)).

Similarly for the NTCP calculation the input data required are No, *K*, TV, *V*_0_, *V*, and EI. To illustrate the application of the code, ten clinical cases planned with different optimization techniques were considered. TCP and NTCP were calculated for each of the iterated treatment plans. The BEEUD-based plan evaluation tool developed in MATLAB environment helps in predicting the TCPs and NTCPs. An attempt was made to correlate the clinical outcomes with the estimated numerical values for cervix cases. The acute toxicities and tumour control were numerically coded based on Common Toxicity Criteria version 2.0 (CTCAE v 2.0) [18].  Table 1 illustrates the numerical scoring criteria.

**Figure 5:**
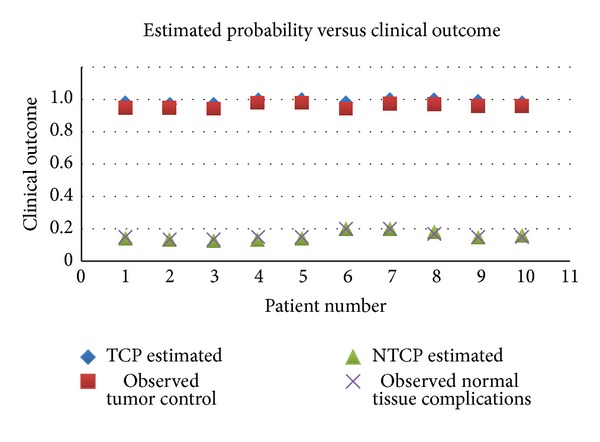
Clinical outcome correlation.

The probability of tumor control and normal tissue complications estimated using evaluation tool are plotted against the observed/numerically coded clinical outcomes (Figure 5). It was found that the observed outcome in the subsequent one-year duration after radiation therapy correlates with the estimated TCPs and NTCPs.

## 4. Discussions

Biologically based treatment planning system (BBTPS) is being put into the clinical use with some limitations such as radiobiological model parameters availability and inadequate clinical response outcome data. But there are many established institutions in the developing nations that use TPSs which are not equipped with these biological evaluation tools. In this context independent software tools were developed for external beam plan evaluation. But many malignancies occurring in organs such as cervix and breast necessitate the brachytherapy treatment also. In such cases clinicians must biologically evaluate the brachytherapy plans also along with EBRT plans. Thus, combined TCP and NTCP values can be estimated which can comprehensively evaluate treatment plans. At the same time the optimal brachytherapy plan can be easily selected based both on dosimetric and biological aspects. The software tool developed could be used to calculate the TCP and NTCP in all the interstitial brachytherapy implants. Currently, the inverse optimization of brachytherapy is based on the dose volume criteria (IPSA, Nucletron). As a future perspective, the radiobiological indices computed from BEEUD concept can also be used as objective function in inverse planning to get optimal plan.

Treatment plan evaluation is often based on the dose volume parameters from DVHs. The limitations in using DVH alone for plan evaluation were clearly being stated by many published reports. Clinical outcomes were said to be correlated with the dosimetric parameters [19]. But it was not clear that which particular *D*-*V* parameter corresponds to the specific outcome (tumour control/normal tissue complications). Typically a DVH curve has different parts which may correlate with different kind of risks. Hence, the probable outcomes in terms of tumour kill and normal tissue toxicities could not be quantified by DVHs. The DVH data does not contain or represent the certain factors which must be taken into account for determining the clinical outcomes.

They are the following:

1. the dose fraction size or number of fractions;
2. elapsed treatment duration (gap between the days);
3. spatial information for locating the hot and cold spots;
4. internal organ motion and deformation which may result in significant variation in volume delineation.

Radiobiological models overcome these difficulties in the plan evaluation. Almost all the commercial TPSs contain only the inbuilt DVH algorithm for evaluation. A prospective analysis of treatment outcome and the selection of best optimal plan for execution might not be possible with DVH tool alone. Hence, there is a need for a sophisticated evaluation tool in addition to DVHs to discriminate between treatment plans. Radiobiological models though available in the literature are less used or often not used because of the mathematics involved. But with the use of computing languages, it is now possible to evaluate the treatment plans comprehensively in radiotherapy clinics.

## 5. Conclusion

A brachytherapy plan evaluation tool to compute radiobiological indices was developed in this research work. Clinicians can utilize the tool to estimate the probabilities of tumour cure and normal tissue complications conveniently. Our module can assist in the evaluation of treatment plans by providing access to outcome predictions. The prediction efficiency of the quality metrics was also clinically assessed in patient cases. The observed clinical outcomes correlated with estimated probabilities. It can be used as a research tool and as a clinical aid.

## Figures and Tables

**Table 1:** Numerical scoring criteria for clinical outcomes.

Grades	Description of clinical outcome	Numerical score
1	Mild, asymptomatic, or mild symptoms, Clinical/diagnostic observations only; intervention not indicated	0.00

2	Moderate; minimal, local, or noninvasive intervention indicated; limiting age-appropriate instrumental ADL*	0.25

3	Severe or medically significant but not immediately life threatening; hospitalization or prolongation of hospitalization indicated; disabling; limiting self-care ADL**	0.50

4	Life-threatening consequences; urgent intervention indicated	0.75

5	Death related to adverse events	1.00

Activities of daily living (ADL).

*Instrumental ADL refer to preparing meals, shopping for groceries or clothes, using the telephone, managing money, and so forth.

**Self-care ADL refer to bathing, dressing, undressing, feeding self, using the toilet, taking medications, and being not bedridden.

**Table 2:** Raw data in excel format.

Differential dose volume graph coordinates	
Dose (cGy)	Volume/dose (cm^3^/cGy)
18.000	0.000
30.000	0.002
42.000	0.052
54.000	0.180
66.000	0.244
78.000	0.210
90.000	0.164
102.000	0.121
114.000	0.100
126.000	0.086
138.000	0.086
150.000	0.110
162.000	0.136
174.000	0.148
186.000	0.141
